# Effect of an intervention in storm drains to prevent *Aedes aegypti* reproduction in Salvador, Brazil

**DOI:** 10.1186/s13071-017-2266-6

**Published:** 2017-07-11

**Authors:** Raquel Lima Souza, Vánio André Mugabe, Igor Adolfo Dexheimer Paploski, Moreno S. Rodrigues, Patrícia Sousa dos Santos Moreira, Leile Camila Jacob Nascimento, Christopher Michael Roundy, Scott C. Weaver, Mitermayer Galvão Reis, Uriel Kitron, Guilherme Sousa Ribeiro

**Affiliations:** 1 Instituto Gonçalo Moniz, Fundação Oswaldo Cruz, Salvador, Bahia Brazil; 2 Instituto de Saúde Coletiva, Universidade Federal da Bahia, Salvador, Bahia Brazil; 3 Universidade Pedagógica de Moçambique, Quelimane, Zambézia Mozambique; 4 Department of Microbiology and Immunology, University of Texas Medical Branch, Galveston, TX USA; 5 Faculdade de Medicina, Universidade Federal da Bahia, Salvador, Bahia Brazil; 6 Department of Environmental Sciences, Emory University, Atlanta, GA USA

**Keywords:** Epidemiology, Entomology, Arboviruses, Insect vectors, Disease vectors, Mosquitoes, *Aedes aegypti*, Storm drain, Catch basin

## Abstract

**Background:**

*Aedes aegypti*, the principal vector for dengue, chikungunya and Zika viruses, is a synanthropic species that uses stagnant water to complete its reproductive cycle. In urban settings, rainfall water draining structures, such as storm drains, may retain water and serve as a larval development site for *Aedes* spp. reproduction. Herein, we describe the effect of a community-based intervention on preventing standing water accumulation in storm drains and their consequent infestation by adult and immature *Ae*. *aegypti* and other mosquitoes.

**Methods:**

Between April and May of 2016, local residents association of Salvador, Brazil, after being informed of water accumulation and *Ae*. *aegypti* infestation in the storm drains in their area, performed an intervention on 52 storm drains. The intervention consisted of placing concrete at the bottom of the storm drains to elevate their base to the level of the outflow tube, avoiding water accumulation, and placement of a metal mesh covering the outflow tube to avoid its clogging with debris. To determine the impact of the intervention, we compared the frequency at which the 52 storm drains contained water, as well as adult and immature mosquitoes using data from two surveys performed before and two surveys performed after the intervention.

**Results:**

During the pre-intervention period, water accumulated in 48 (92.3%) of the storm drains, and immature *Ae*. *aegypti* were found in 11 (21.2%) and adults in 10 (19.2%)*.* After the intervention, water accumulated in 5 (9.6%) of the storm drains (*P* < 0.001), none (0.0%) had immatures (*P* < 0.001), and 3 (5.8%) contained adults (*P* = 0.039). The total number of *Ae*. *aegypti* immatures collected decreased from 109 to 0 (*P* < 0.001) and adults decreased from 37 to 8 (*P* = 0.011) after the intervention. Collection of immature and adult non-*Aedes* mosquitoes (mainly *Culex* spp.) in the storm drains also decreased after the intervention.

**Conclusion:**

This study exemplifies how a simple intervention targeting storm drains can result in a major reduction of water retention, and, consequently, impact *Ae*. *aegypti* larval populations. Larger and multi-center evaluations are needed to confirm the potential of citywide structural modifications of storm drains to reduce *Aedes* spp. infestation level.

## Background

Arboviral infections are an important public health problem in the tropics and subtropics, both because of their high incidence and due to the severe manifestations that may follow (e.g., dengue hemorrhagic fever and dengue shock syndrome, chikungunya chronic arthritis, and Zika congenital malformations). As no effective antivirals or affordable vaccines are available, control of mosquito vector populations remains the primary tool for reducing virus transmission and disease. However, reducing the infestation levels of *Aedes* (*Stegomyia*) spp. mosquitoes that serve as vectors for dengue (DENV), Zika (ZIKV) and chikungunya viruses (CHIKV), and potentially yellow fever virus, remains a challenge worldwide [1].

Since 2015, Brazil has been in the spotlight due to the large ZIKV epidemic and ensuing congenital and neurological complications [2]. Brazil is also the country that reports the highest number of dengue cases in the world [3], and in 2016 was responsible for 76% of the 351,300 suspected cases of CHIKV infections reported in the Americas [4]. The Brazilian National Dengue Control Program has emphasized education of the population to eliminate household containers that may hold water and serve as oviposition and larval development sites, and conducts entomological surveys to detect and eliminate these sites or treat them with larvicides. Campaigns typically only target the intra-domiciliary larval development sites, neglecting potential sites located in public spaces. However, growing evidence from various American countries and Australia have pointed to urban drainage structures, such as storm drains, that hold rainfall and runoff water, as a productive environment for *Aedes* spp. immatures [5–8].

**Fig. 1:**
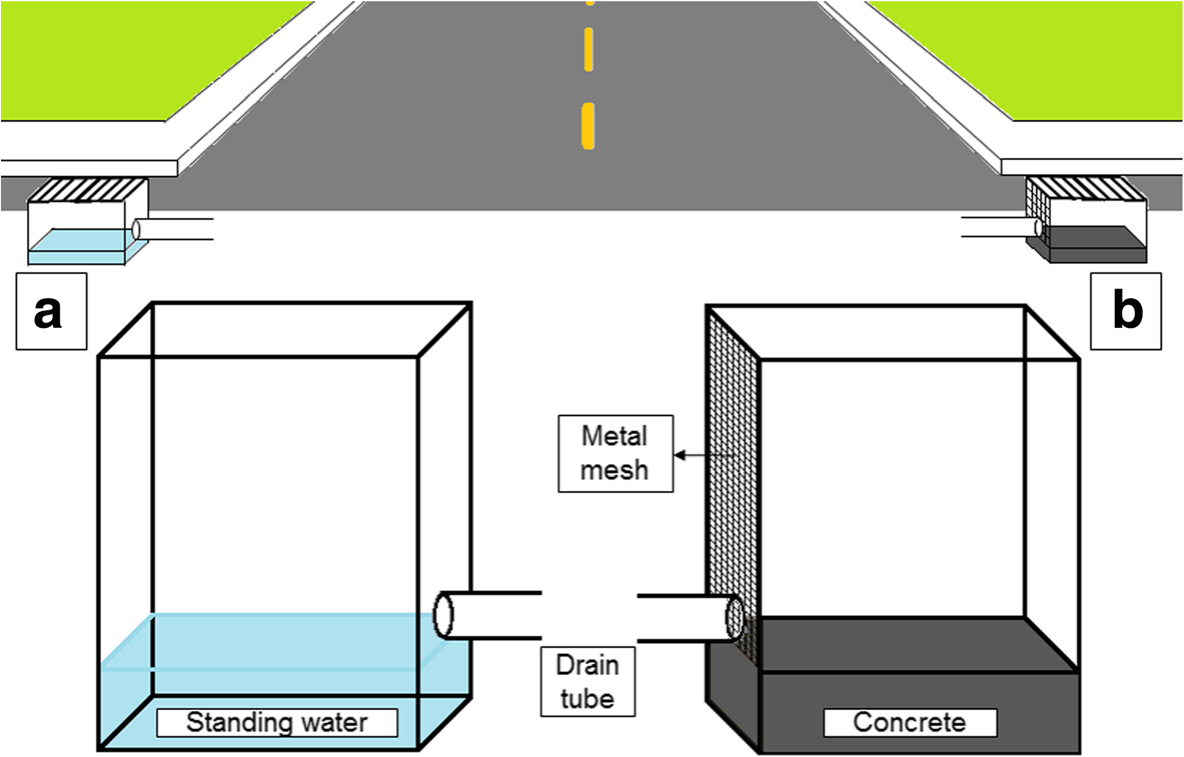
Scheme of Piatã storm drains before (**a**) and after (**b**) the intervention. In **a**, standing water is shown accumulated in the bottom of the storm drain. In **b**, the bottom of the storm drain is filled with concrete, allowing water outflow through the drain tube, and metal mesh is installed to prevent debris from clogging into the drain tube

In Salvador, northeastern Brazil, we systematically surveyed storm drains in four different study sites during 2015, and found accumulated water in ~50% of the 241 inspections [5]. A total of 468 adults and immature specimens were collected: 148 *Ae*. *aegypti*, 79 *Ae*. *albopictus*, and 241 non-*Aedes* mosquitoes (mainly *Culex* spp.). After being informed of the results of our surveys, the community leaders from one of the study sites (Piatã community) decided to perform an out-of-pocket intervention targeting the 52 storm drains located in their community to prevent accumulation of water. The intervention consisted of placing concrete in the bottom of the storm drains, aiming to raise the bottom level equal to that of the outflow draining tube, and thus prevent standing water (Fig. 1). In addition, a metal mesh was installed over the outflow drain tube to trap debris and prevent it from entering the drain and restricting water flow. The intervention was conducted during March-April 2016. To evaluate the effect of this intervention, we conducted two additional surveys of these 52 storm drains, and compared our findings with the results of the two surveys that we had performed prior to the intervention.

## Methods

Details of the Piatã study site and of the pre-intervention storm drain surveys were described previously [5]. Piatã, a residential, high socioeconomic level neighborhood with ~30,000 inhabitants, is composed of several gated communities comprising individual homes with yards. Its appearance is similar to that of many U.S. suburbs and the structure of the Piatã storm drains is similar to that of other places in Salvador and Brazil: a series of concrete rectangle containers below the street gutters, each container ~100 cm long, ~30 cm wide, and ~50 cm deep, covered with a concrete or metal grate, and with discharge pipes exiting near the bottom. Piatã is adjacent to the Atlantic coast, and the Piatã study site is ~200 m from Piatã beach. Administratively, the Piatã neighborhood is part of the Sanitary District of Itapuã and the incidence of reported cases of dengue, Zika and chikungunya virus infection in this Sanitary District were 172, 399, and 41.7 cases per 100,000 inhabitants, respectively, in 2015, and 127.7, 15.7 and 14.2 cases per 100,000 inhabitants, respectively, in 2016.

To assess the effect of the intervention, all 52 Piatã storm drains that underwent the intervention were surveyed four times, twice before the intervention (March 10 or 13, 2015; and March 28, 2015) and twice after the intervention (April 21, 2016; and May 21, 2016). The same research team conducted the pre- and post-intervention surveys, using the same methods: adult mosquitoes captures using Prokopack aspirators [9], estimation of accumulated water volume (by measuring and multiplying the height, width and length of the storm drain water accumulation), and collection of 1 l of the surface water (or the total volume of water when < 1 l was available), to search for and collect mosquito immatures. We reared immatures for up to 10 days to allow for development to adults and identified adult mosquitoes (either aspirated or reared from larvae/pupae) with a dissecting scope as *Ae*. *aegypti*, *Ae*. *albopictus* or non-*Aedes* mosquitoes.

### Statistical analysis

We used absolute and relative frequencies to describe the collected data. Given that we performed two repeated surveys in the same storm drains both before and after the intervention, we aggregated the data from the two pre-intervention surveys and did the same for the two post-intervention surveys. Then, we used MacNemar’s test for paired data to assess whether the proportion of storm drains that contained water, as well as adult and immature mosquitoes, differed between the pre- and post-intervention surveys. Finally, to compare the average volume of water in the storm drains that contained water and the counts of captured adult and immature mosquitoes on the species level before and after the intervention, we used the Wilcoxon signed-rank test. Differences were considered statistically significant at *P* < 0.05.

## Results

**Fig. 2:**
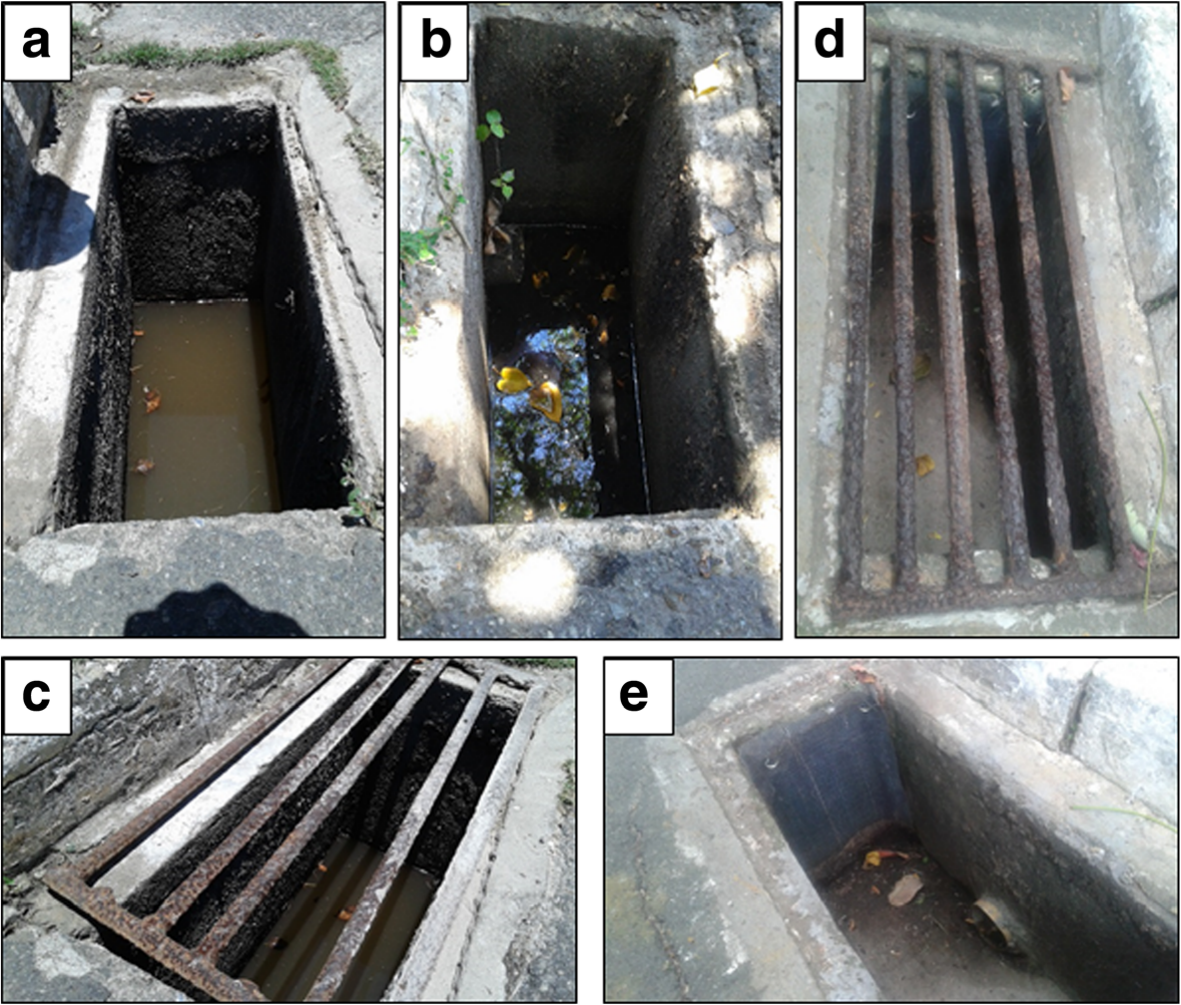
Typical storm drains surveyed in Piatã neighborhood in the 2015 pre-intervention period (**a**, **b**, **c**) and in the 2016 post-intervention period (**d**, **e**), in Salvador, Brazil

The proportion of storm drains with residual water was reduced by nearly an order of magnitude in the post-intervention surveys compared to the pre-intervention surveys (Table 1). The aggregated data from the surveys conducted in the 52 storm drains before the intervention showed the presence of accumulated water in 48 (92.3%), while the aggregated data from the surveys conducted after the intervention indicated water accumulation in only 5 (9.6%; *χ* ^2^ = 43.0, *df* = 1, *P* < 0.001) (Figs. 1 and 2). The average accumulated water volume in the storm drains that contained water was reduced from 41.6 l in the pre-intervention surveys to 1.6 l in the post-intervention surveys (*Z* = 2.0, *P* = 0.043), even though the average total precipitation during the week prior to the two pre-intervention surveys was actually higher than during the week prior to the two post-intervention surveys (*Z* = -6.5, *P* < 0.001).

**Table 1:** Findings from entomological surveys performed in 52 storm drains, before and after an intervention to prevent water accumulation in Salvador, Brazil

Survey characteristics	Pre-intervention (Year: 2015)			Post-intervention (Year: 2016)			*P* ^a^
	First survey	Second survey	Total	First survey	Second survey	Total	
Survey date (day/month)	10 & 13/Mar	28 & 29/Mar	Mar	21/Apr	21/May	Apr-May	
Average precipitation (mm) during 7 days prior to survey	2.7	60.3	31.5	23.2	66.4	44.8	< 0.001
No. (%) of storm drain with accumulated water	38 (73.1)	48 (92.3)	48 (92.3)	2 (3.9)	4 (7.7)	5 (9.6)	< 0.001
Average volume (l) of water	32.2	51.0	41.6	0.45	2.7	1.6	0.043
No. (%) of storm drain containing larvae/pupae of							
*Aedes aegypti*	11 (21.2)	1 (1.9)	11 (21.2)	0 (0)	0 (0)	0 (0)	< 0.001
Non-*Aedes* mosquitoes	19 (36.5)	2 (3.8)	19 (36.5)	0 (0)	1 (1.9)	1 (1.9)	< 0.001
No. (%) of storm drain containing adults of							
*Aedes aegypti*	8 (15.4)	2 (3.8)	10 (19.2)	3 (5.8)	0 (0)	3 (5.8)	0.039
*Aedes albopictus*	0 (0)	0 (0)	0 (0)	1 (1.9)	0 (0)	1 (1.9)	1.00
Non-*Aedes* mosquitoes	12 (23.1)	8 (15.4)	13 (25.0)	2 (3.8)	0 (0)	2 (3.8)	< 0.001
Number of larvae/pupae collected							
*Aedes aegypti*	108	1	109	0	0	0	< 0.001
Non-*Aedes* mosquitoes	161	6	167	0	42	42	< 0.001
Number of adults captured							
*Aedes aegypti*	35	2	37	8	0	8	0.011
*Aedes albopictus*	0	0	0	1	0	1	0.317
Non-*Aedes* mosquitoes	16	25	41	4	0	4	< 0.001

*Aedes albopictus* larvae or pupae were not detected in any of the surveys. Non-*Aedes* mosquitoes were mainly *Culex* spp. mosquitoes

^a^ *P*-values for the comparison between pre- and post-intervention totals using MacNemar’s test or Wilcoxon signed-rank test

Following the intervention, we found lower proportions of storm drains containing immatures of either *Ae*. *aegypti* (from 21.2% to 0.0%; *χ* ^2^ = 11.0, *df* = 1, *P* < 0.001) or non-*Aedes* mosquitoes (from 36.5% to 1.9%; *χ* ^2^ = 18.0, *df* = 1, *P* < 0.001) (Table 1). We found comparable reductions in the proportions of storm drains containing adult *Ae*. *aegypti* (from 19.2% to 5.8%; *χ* ^2^ = 5.4, *df* = 1, *P* = 0.039) and adult non-*Aedes* mosquitoes (from 25.0% to 3.8%; *χ* ^2^ = 11.0, *df* = 1, *P* < 0.001) (Table 1). None of the storm drains surveys detected immature *Ae*. *albopictus*, and we only captured a single adult *Ae*. *albopictus* in one (1.9%) storm drain in one post-intervention survey (Table 1).

The aggregated numbers of both immatures and adults of *Ae. aegypti* and non-*Aedes* mosquitoes collected during the pre-interventions surveys was also greater than post-intervention (Table 1). The total numbers of collected specimens in the pre- and post-intervention surveys were, respectively, 109 and 0 immature *Ae*. *aegypti* (*Z* = 3.3, *P* < 0.001), 167 and 42 immature non-*Aedes* (mostly *Culex* spp.) (*Z* = 3.8, *P* < 0.001), 37 and 8 adult *Ae*. *aegypti* (*Z* = 2.6, *P* = 0.011), and 41 and 4 adult non-*Aedes* mosquitoes (mostly *Culex* spp.) (*Z* = 3.6, *P* < 0.001).

## Discussion

Overall, the results presented here show that a simple, direct intervention targeting storm drains resulted in large reductions in frequency and amount of residual water, as well as in the numbers of immature and adult *Ae*. *aegypti* and of non-*Aedes* mosquitoes therein. Adult mosquitoes captured inside storm drains may include adults that emerged from pupae in the same storm drains, females in an immediate pre- or post-oviposition stage, or adults resting in the sheltered environment. It is possible that the intervention not only eliminated the first two, but also reduced the suitability of the storm drains as resting sites via reductions in humidity.

The importance of subterranean habitats for *Aedes* mosquito proliferation was also reported from Australia, México and Singapore [6, 7, 10]. In addition, a study reporting the effect of an intervention based on regular application of larvicides in storm drains in Colombia showed a reduction in the number of dengue cases following the intervention [11]. Our prior study [5] and the study presented here lend further support for the role of water drainage structures in urban settings for the proliferation *Aedes* mosquitoes, and demonstrate that interventions targeting storm drains can render them unsuitable for mosquito oviposition and larval development.

It is worth noting that residents of the community spontaneously reported that mosquito infestation levels and annoyance were lower after the intervention. Based on this information, we interviewed ten residents of different households (out of 60 houses in the community) in order to better ascertain this observation. To reduce the potential for information bias, we choose an interviewer who had not participated in the storm drain surveys, and we did not mention the intervention during the interview. Seven of the ten interviewed residents reported a reduction in the number of mosquitoes in their house when asked about any change in mosquito numbers in the preceding month (post-intervention period) and four of them (57.1%) attributed the reduction to the intervention in the storm drains. These almost anecdotal observations are from a small sample of individuals, but are in agreement with the hypothesis that storm drains serve as cryptic oviposition sites in urban settings, and contribute to mosquito numbers in certain communities.

Prior interventions targeting storm drains for prevention of arboviral diseases were based on regular application of larvicides, such as pyriproxyfen [11] or insect growth regulators (IGRs). However, because these structures are designed to drain water from public areas, larvicides or IGRs are typically washed away following rainfall, and thus their effect on the mosquito’s reproductive cycle is temporary. Additionally, chemical interventions need to be re-applied over the years, requiring constant visits and material purchases. In contrast, interventions that permanently modify the configuration of the storm drains’ structure, such as the one we described in this study, may provide a long-term alternative by reducing water accumulation and subsequent larval proliferation. Yet, it is important to highlight the need for continuing searches and removal/treatment of diverse larval sites, because when an intervention targets highly productive containers, females that would have oviposited there may switch to alternative water containers that were less productive before [12]. Searching for non-traditional larval habitats is increasingly important as *Aedes* mosquitoes continuously adapt to the ever-changing urban environment.

Table 1 suggests that rainfall may be conditioning mosquito larval populations in the storm drains studied. On our previous work, in which we reported that storm drains serve both as larval development and adult resting sites, we identified that more than 50 mm of rain on the 7 days before the storm drain survey lowered the odds of finding mosquito adults or immatures by 80% [5]. Thus, the findings of entomological surveys in these kinds of cryptic sites might be strongly affected by the preceding precipitation. To account for this potential source of bias, we performed two surveys pre and post-intervention (for a total of four surveys) to diminish the impact that precipitation could have on the average counts of mosquito on each visit. Table 1 also suggests that in the pre-intervention phase of the study, despite 92% of the storm drain having standing water, only 21% were infested. This may be explained by the high rainfall that occurred during the pre-intervention phase of the study. Rain flushes larvae out of the storm drain, thus lowering the odds of finding immature mosquitos in storm drains immediately following rainfall. Some storm drains may be more prone to mosquito infestation, such as those located under trees (which provide shade and lower temperatures), than those exposed to direct sunlight. Although not all storm drains that hold water will be used by mosquitoes for larval development, even if only a small percentage (10 or 20%) does, the volume of water they hold, average of 42 l [5], and its ubiquity in the urban environment allow for the potential production of a non-negligible number of mosquitoes.

In this study, the community organized itself to conduct the intervention in the storm drains. As a gated community, a committee of residents is responsible for management and administration of the area. With the information that storm drains in the community accumulated water, the committee decided to intervene with support from the residents. No approval from city agencies was needed, since the intervention did not change the function of the storm drains. Modifying all storm drains in the community took less than one week by two employees, at an estimated cost (personnel plus materials) of approximately U$18.00 (R$50.00) per storm drain. The total cost of the intervention was shared among all residents in the community.

This study serves as a demonstration that a simple yet effective intervention on storm drains that accumulate water is feasible. Scaling this action to an entire city would require inter-sectorial involvement to meet diverse challenges, such as different drain designs across the city, societal acceptance of the action and human resources to conduct the intervention. We recommend that cities interested in conducting such an intervention first assess whether their storm drains also allow for water accumulation and mosquito proliferation and determine if the intervention here described is the best solution to the problem in their specific context.

The main limitation of our study is the lack of a control community, which would allow for a comparison of our findings with a setting where no intervention was implemented. However, although we cannot completely rule out that the observed reductions in storm drain accumulated water and mosquito infestation were due to seasonal fluctuations, this is unlikely because: (i) the reduction in the proportion of storm drains containing water and the total volume of water inside decreased after the intervention despite greater rainfall during that period; (ii) we conducted two surveys both before and after the intervention, and the findings were consistent. Larger, multi-center, controlled studies are needed to determine whether our findings can be used to justify citywide (or at least neighborhood level) structural modifications of storm drains as an effective measure to reduce *Aedes* spp. infestation levels.

## Conclusions

In addition to describing a successful intervention targeting storm drains, our study elucidates a pertinent experience in which residents of the community studied organized themselves to conduct an intervention after we informed them about the findings of an earlier study that we performed in their community [5]. This exemplifies how communication of research findings may empower community groups to mobilize and improve the environment in order to reduce health risks. As a follow-up to this study, we are working with Salvador public officials to evaluate the role of storm drains in other parts of the city, and to design further interventions in areas where storm drains with residual water are common.

## Abbreviations

CHIKV: Chikungunya virus
DENV: Dengue virus
IGRs: Insect growth regulators
ZIKV: Zika virus
